# Enhancing Tissue Equivalence in ^7^Li Heavy Ion Therapy with MC Algorithm Optimized Polymer-Based Bioinks

**DOI:** 10.3390/jfb14120559

**Published:** 2023-11-25

**Authors:** Fatih Ekinci, Koray Acici, Tunc Asuroglu

**Affiliations:** 1 Institute of Nuclear Sciences, Ankara University, 06100 Ankara, Turkey; fatihekinci@ankara.edu.tr; 2 Artificial Intelligence and Data Engineering, Ankara University, 06100 Ankara, Turkey; kacici@ankara.edu.tr; 3 Faculty of Medicine and Health Technology, Tampere University, 33014 Tampere, Finland

**Keywords:** polymeric biomaterials, lithium-ion therapy, Bragg cure, recoil, MC algorithm

## Abstract

The unique physical properties of heavy ion beams, particularly their distinctive depth–dose distribution and sharp lateral dose reduction profiles, have led to their widespread adoption in tumor therapy worldwide. However, the physical properties of heavy ion beams must be investigated to deliver a sufficient dose to tumors without damaging organs at risk. These studies should be performed on phantoms made of biomaterials that closely mimic human tissue. Polymers can serve as soft tissue substitutes and are suitable materials for building radiological phantoms due to their physical, mechanical, biological, and chemical properties. Extensive research, development, and applications of polymeric biomaterials have been encouraged due to these properties. In this study, we investigated the ionization, recoils, phonon release, collision events, and lateral straggle properties of polymeric biomaterials that closely resemble soft tissue using lithium-ion beams and Monte Carlo Transport of Ions in Matter simulation. The results indicated that the Bragg peak position closest to soft tissue was achieved with a 7.3% difference in polymethylmethacrylate, with an average recoils value of 10.5%. Additionally, average values of 33% were observed in collision events and 22.6% in lateral straggle. A significant contribution of this study to the existing literature lies in the exploration of secondary interactions alongside the assessment of linear energy transfer induced by the ^7^Li beam used for treatment. Furthermore, we analyzed the tissue-equivalent properties of polymer biomaterials using heavy ion beams, taking into account phonon release resulting from ionization, recoils, lateral straggle, and all other interactions. This approach allows for the evaluation of the most suitable polymeric biomaterials for heavy ion therapy while considering the full range of interactions involved.

## 1. Introduction

Heavy ion therapy currently stands as the prevailing approach in tumor treatment [1,2,3]. These ions exhibit a high linear energy transfer (LET) as they deposit a greater amount of energy (keV) per unit distance (μm) than photons at the Bragg peak [4]. Despite these inherent advantages, recent years have brought to light the limitations of ion species once deemed ideal, such as protons and carbon ions, for therapeutic purposes. Consequently, there has been a growing emphasis on research involving alternative ion species, particularly grounded in clinical observations [5]. In essence, the physical and biological attributes of an ion beam are contingent upon the mass number of the respective clinical outcome particle associated with each ion. Commonly utilized ions in both clinical practice and theoretical exploration encompass protons (P), carbon (C), helium (He), lithium (Li), beryllium (Be), boron (B), and nitrogen (N) ions, all of which have elicited significant interest [6,7,8]. Nevertheless, definitive evidence regarding the suitability of these ion species for clinical therapy remains elusive [9].

To this end, the application of ion beams within the treatment planning system has been instrumental in formulating realistic treatment plans and evaluating potential advantages [10]. Extensive investigations have been conducted on ion beams with varying mass numbers, including P, He, B, C, N and O ions, both from clinical and theoretical perspectives [1,6,11]. It has been posited that an optimal ion beam may exist for each specific combination of ion type and tissue [1,2,4]. Additionally, comprehensive assessments of interactions, such as ionization and recoils, have been carried out across a spectrum of ion beams up to mass number 16 [1,11]. In these investigations, ion beams with a mass number of 8 or greater demonstrated similar effectiveness for targets situated in proximity to the surface [1]. Nonetheless, uncertainties persist in identifying the most suitable ion beam for therapeutic applications, prompting ongoing research endeavors. Consequently, the exploration of novel ion beams, such as ^7^Li, within phantoms composed of diverse biomaterials has emerged as a pivotal pursuit. Lithium, a significant metallic element globally, is naturally present at concentrations of 20–60 ppm in crustal rocks, 0.2 ppm in seawater, and 0.001–0.01 mM in mammals [12]. Notably recognized for its unique attributes, including substantial heat capacity, high redox potential, and efficient electrochemically active properties [13]. The ^7^Li heavy ion is an ion that falls between hydrogen (H) and helium (He) on the periodic table. Consequently, a similar ionization value can be obtained with less energy compared to helium, which presents a significant advantage in heavy ion therapy. This characteristic categorizes it as an intermediate ion primarily because it is believed to exhibit less lateral scattering than hydrogen.

Owing to the high LET associated with heavy ion beams, even millimeter-scale deviations in the Bragg peak on the target have been observed to result in damage to healthy tissues [14]. Consequently, it becomes paramount to precisely determine the range of ion beams utilized for therapeutic purposes within the tissue context [14]. As such, the utilization of Monte Carlo (MC)-based simulation systems for precise calculations is of utmost significance prior to embarking on heavy ion treatment planning [15]. Simulation programs such as MC Transport of Ions in Matter (TRIM), which are used in heavy ion therapy, are similar to GEometryANd Tracking 4 (GENAT4), FLUktuierendeKAskade (FLUKA), and the Particle and Heavy Ion Transport code System (PHITS), and they are readily available.

GEANT4 and FLUKA are employed in simulating the transport and interactions of high-energy particles. They encompass various physical processes, including nuclear, hadronic, electromagnetic, and accelerator physics. These programs provide detailed simulations of charged particle interactions within matter, covering a wide range of physics. They have been developed by organizations like the Conseil Européen pour la Recherche Nucléaire and the Istituto Nazionale di Fisica Nucleare [16,17].

PHITS is specifically used in simulating the transport and interactions of high-energy particles, including various physical processes like nuclear, hadronic, electromagnetic, and accelerator physics. It offers detailed simulations of charged particle interactions within matter and has been developed by the Japan Atomic Energy Agency [18].

TRIM specializes in ion implantation and similar applications, allowing for accurate calculations of ion range and energy loss. It can swiftly determine the range of a specific ion in a particular material, which can be vital for making rapid decisions. Thanks to its user-friendly interface, determining the ion range in specific materials can be straightforward [8,16,17,18]. TRIM has been designed specifically for use in semiconductor technology, material science, and certain microelectronics applications where it is widely employed. It permits users to adjust ion–material interactions using specific parameters, facilitating a better understanding of interactions in layered structures and leading to more precise results in specialized applications. These advantages make TRIM particularly preferred for specific application areas [1,16,17,18]. Given the distinctive layered structure of the human body, which is composed of various biomaterials, each possessing distinct properties, deviations in the direction of ion beams become a critical consideration in clinical applications [14]. The selection of an appropriate heavy ion beam with minimal aberrations assumes great importance in safeguarding the healthy tissues surrounding the target tumor. To this end, it is imperative that the deviation range of the chosen heavy ion beam be rigorously verified with submillimeter precision through either phantom studies or simulations [14].

The physical, mechanical, radiological attenuation, and scattering properties of phantom materials used to replicate target tissue in clinical ion treatments play a pivotal role [19]. Particularly, radiological investigations employing phantom models composed of biomaterials closely resembling soft tissue are essential [20]. One such biomaterial is polymethylmethacrylate (PMMA) [21]. PMMA’s fundamental molecular composition is C_5_H_8_O_2_, boasting a bulk density of 1.19 g/cm^3^ and a stopping power relative to water at 1.156 [21]. Owing to these characteristics, PMMA has gained wide utilization in radiotherapy for dose measurements and calibration experiments, effectively serving as a surrogate for soft tissue [21].

In this study, given the absence of an experimental ^7^Li ion beam line, we employed the MC Transport of Ions in Matter (TRIM) simulation system to contribute valuable insights to the field. The radiological properties of the ^7^Li ion beam were meticulously explored in water, soft tissue, Polystyrene, Resin, Epoxy and PMMA biomaterials, with the objective of advancing biophysical dosimetry methodologies and establishing robust criteria for radiological research. Moreover, we conducted a comprehensive assessment of ionization, recoil, phonon release, collision events (CE), and lateral straggle (LS) specific to the ^7^Li ion beam, representing a novel aspect of this study based on the biomaterial type. Another innovative aspect of this research involved comparing these results with both water as a calibration material and actual tissue. Thus, to validate the radiological findings pertaining to the ^7^Li ion beam, data were reported using phantoms crafted from three distinct biomaterials in line with the experimental methodology.

## 2. Material and Method

**Figure 1:**
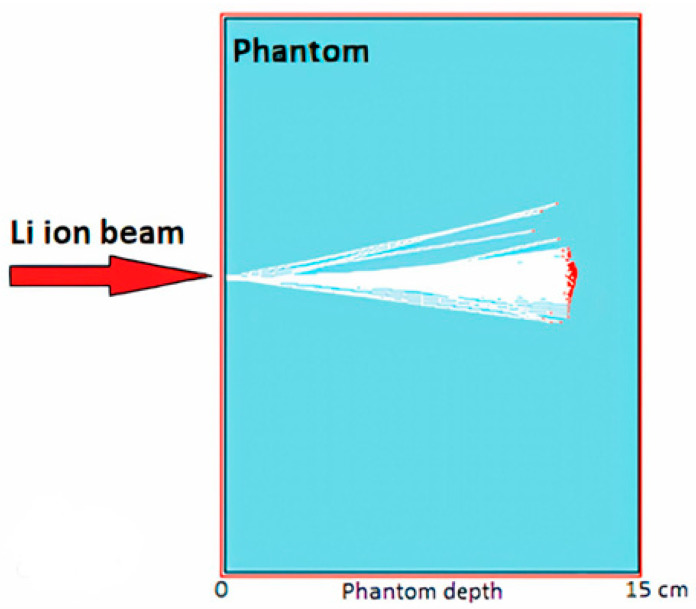
Representation of the range and LS of the ^7^Li beam in the PMMA phantom created in the TRIM simulation system.

In this study, a simulation was made using phantoms made of different materials used in biomedical applications. In the simulation, a ^7^Li ion beam was used, and phantoms made of biomaterials such as PMMA and water were bombarded with a ^7^Li ion pen beam with a particle count of 10^5^ at therapeutic energies. The results obtained from the phantom created using the MC TRIM method are discussed in relation to similar studies in the literature. The single-layer phantom shown in Figure 1, with a depth of 15 cm and a surface of 8 cm, was constructed using three biomaterials.

The MC TRIM program used in this study is a program that can operate in a wide range of ion types and energies, and parameters such as beam incidence and particle number can be adjusted. The program can create the selected phantom type, shape and number of layers at the desired level. In addition, the program can calculate the energy loss processes of ions, such as damage to the target, scattering, ionization, cavities in the crystal structure of selected biomaterials, phonon production and recoil [22]. The program can track and record in detail the atomic cascades of the biomaterials that make up the phantoms on the target. It can also calculate all the kinetic events in the target phantom of the ^7^Li ion beam. This program is a widely used tool for creating phantoms and analyzing radiation effects for biomedical applications [23,24].

Polystyrene (PS) has good transparency, lack of color, ease of manufacture, thermal stability, low specific gravity (1.04–1.12 g/cm^3^) and relatively high modulus [25]. PS is resistant to impact strength and environmental stress cracking [26]. PS blood dialyzers are used in a wide range of medicinal applications, such as diagnostic test kits [27].

PMMA polymer boasts an extensive molecular structure owing to its covalent bond chain configuration [25]. These elongated chains are bound together by secondary forces like van der Waals and hydrogen bonds or by primary covalent forces through crosslinks between chains [25]. However, when the chains become exceedingly long, they fail to achieve complete crystallization, resulting in a semi-crystalline structure. Natural polymers, such as semi-crystalline polysaccharides and proteins, are also produced through condensation polymerization. PMMA, widely employed in biomedical applications, offers a range of advantages, including high transparency, durability, chemical resistance, heat resistance, and processability, rendering it a valuable asset in medical applications such as radiotherapy [25,26].

PMMA, with its amorphous nature (Tg: 105 °C and density: 1.15–1.195 g/cm^3^), possesses attributes like a high refractive index, light transmittance, and weather resistance, enhancing its biocompatibility [25,26]. Its ease of processing using conventional instruments or plasma treatment further extends its utility in various medical applications, including blood pumps, reservoirs, contact lenses, dentures, maxillofacial prostheses, and bone cement for joint prosthesis fixation. Additionally, PMMA plays a crucial role as a phantom material for evaluating the dosimetric properties of biomaterials in radiotherapy [25,27].

Table 1 provides data on atomic percent (%), mass percent (%), atomic number density (×10^22^ atoms/cm^3^), mass density (g/cm^3^), displacement (eV), binding energy (eV), and surface energy (eV) for these biomaterials within the MC TRIM simulation system. These properties listed in Table 1 are known to exert significant influence on ionization, recoils, phonon production, and LS, all of which are critical factors in radiological interactions.

The crystal structure determines the physical properties (e.g., hardness, density, thermal conductivity) and chemical reactivity of a substance. Therefore, understanding the crystal structure of a material is important for comprehending its behavior and characteristics. Polymers generally do not have a crystalline structure. The reason for this is that polymer molecules tend to come together in an irregular and random manner [28,29]. A crystalline structure is an arrangement where atoms or molecules are organized in a specific order and symmetry. The molecules of polymers are typically long and chain-like, which makes it difficult to achieve a crystalline arrangement. However, under certain conditions, regular crystalline structures can form in specific regions of polymers [29,30]. These regions are referred to as crystalline injection zones. These areas allow for the formation of a crystalline structure, particularly under specific conditions such as certain temperatures and pressures. In general, most polymers are amorphous, meaning they do not have a regular crystalline structure. This property allows polymers to be elastic and flexible, but it also leads to lower hardness values [28,29,30]. In this study, the crystalline injection zones formed during the preparation of the biomaterial by ^7^Li heavy ions and the damage caused in the polymer chains were investigated. Thus, the displacements occurring in the atoms that constitute these structures were examined.

The main innovation in this study is the parameters of recoils and CE. The model, commonly known as the NRT (Norgett, Robinson, and Torrens) [31] model, is expressed in Equation (1):

*N_v_* = *ρ N_A_*Φ*_v_* (1)

here, *ρ* is the density of the target material, *N_A_* is the Avogadro number, and Φ*_v_* is the energy of the primary ion and a constant valid for the target material [31]. Here, *N_v_*; displacement primary impact atom number, *ρ*; the density of the target material is *N_A_*: Avogadro number and Φ*_v_*; energy can be expressed as the number of collisions per unit area (also known as “collision frequency” for short) and the number of collisions per unit area per unit time and per unit area [31]. The *ρ*, *A* and Φ*_v_* parameters in this formula can be calculated using the MC TRIM program. The use of this theory is particularly important in examining the damage caused by high-energy ions [31,32]. The Norgett, Robinson, and Torrens (NRT) theory is a model developed to analyze energy losses and transfers in crystal structures commonly applied in techniques such as electron microscopy. Polymers, on the other hand, are typically characterized by irregular and amorphous molecular structures. Consequently, lacking a direct crystalline lattice, the NRT theory was not originally designed for polymers. However, similar theoretical approaches and experimental methodologies can be employed to investigate electron interactions and energy losses in polymer materials. Such endeavors are pivotal in comprehending the electronic, mechanical, and optical properties of polymers, ultimately contributing to the advancement of their industrial and scientific applications [33]. Recoils and CE parameters calculate the displacement of target material atoms interacting with ions, as well as the formation of cavities in the crystal structure and their effect on ionization events. Therefore, these parameters help to understand the effects of ions on the target material in detail and allow more accurate modeling of the effects of ions used in radiotherapy and other applications of radiation in the biomedical field [32]. Additionally, the number of displaced atoms *N_v_* in Equation (2):

(2) $$N_{v}\left\{\begin{array}{cc} 0 & E_{v}<E_{d}\\ 1 & E_{d}\leq E_{v}<2.5E_{d}\\ \frac{0.8E_{v}}{2E_{d}} & E_{v}\geq 2.5E_{d} \end{array}\right\}$$

Equation (2) is part of the Kinchin–Pease theory, which is a method in which the energies of the ions on the target material and the displacement energies of the primary impact are used. The Kinchin–Pease theory is a mathematical modeling method for the energy losses resulting from the interactions of charged particles, predominantly electrons, with matter. This theory describes the statistical distribution of energy losses and takes into account various mechanisms of energy loss. Polymers are typically large molecules with irregular and amorphous structures. These characteristics make the direct application of the theory challenging, as this theory was originally developed for crystalline structures. However, similar theoretical approaches and experimental methods concerning electron interactions and energy losses in polymers have been successfully employed for their characterization. The theory can be utilized as a tool to understand and characterize energy losses in polymer materials. Such studies are crucial for gaining insights into the electronic, mechanical, and optical properties of polymers [34]. *N_v_* is the number of displacement atoms; *E_v_* is the damage energy of the ion; *E_d_* is the threshold displacement energy; and 2*E_d_* denotes twice the threshold displacement energy. Additionally, *E_v_* is the kinetic energy of the ion, and 0.8*E_v_*/2*E_d_* is the scattering energy of the ion [32].

Another concept emphasized in this study is LS, which refers to the dispersion that occurs as a result of the change of direction of ions as they pass through the target material [35]. As a result of LS, the projection ranges and interaction distances of the ions change and, therefore, may cause changes in the material properties [35]. LS is an important parameter to understand the effect of ions on the material and to characterize the material. In this concept, *x_i_* is the projection range of the ion “*i*” on the *x*-axis; Σ*_i_ x_i_* = sum of ion projection ranges; Σ*_i_x_i_*/*N* = mean projection range (*R_p_*) of *N* ions and <*x*> = average projection range of all ions. The transverse coordinate “*y*” is treated in the same way, only the distance in the *XY* plane [35]. Thus, LS:

*σ* = [(Σ*_i_x_i_*^2^)/*N* − *R_p_*^2^]^1/2^ = <(Δ*x_i_*)^2^>^1/2^ (3)

This expression gives information about the symmetry property of the gap distribution for a normal bullet sent by the ion beam. This symmetry property means that it will be the same along a plane perpendicular to the projectile (i.e., *R_y_* = 0). This means that the average lateral reflected range of the projectiles sent by the ion beam is zero. Additionally, averaging the calculated ranges along the *Y* and *Z* axes is a way to improve the accuracy of the calculated results. This method is used to obtain a more accurate result by averaging the calculated ranges. These expressions provide information about the methods used to increase the accuracy of the calculations for a normal bullet sent by the ion beam [35].

*σ_y_* = [Σ*_i_*((|*y_i_*| + |*z_i_*|)/2)^2^/*N*]^1/2^ (4)

Standard Deviation (S.D.) is a crucial statistical metric in the literature, measuring the spread of values around the mean in a dataset. In this study, it has been used to determine and analyze variability in the datasets. A low standard deviation indicates that the data points are more tightly clustered around the mean, while a high standard deviation indicates that the data are distributed over a wider range. Additionally, a different tool, Average, has been applied to each dataset. Thus, the aim was to identify the general trend of the datasets and comprehend the typical value of data points. It is well-known that the mean serves as a robust tool in the literature for understanding the overall structure and trends of datasets, playing a significant role in decision-making processes. In this study, the term Standard Deviation (SD) is employed to elucidate the distribution of interactions between Li heavy ions and the same biomaterials at different energy levels. When heavy ions naturally interact with biomaterials, the frequency and magnitude of these interactions change proportionally within the energy range from entry to the Bragg peak. These variations are discernible in the tables and graphs we present. However, due to the inherent nature of the Monte Carlo (MC) system, margins of error exist. These margins can be observed by comparing the SD values of interactions. Calculating SD in different biomaterials at the same energy level is meaningless, given the distinct chemical and physical properties of each biomaterial. For all these reasons, it is emphasized that the obtained SD value is not directly correlated with energy. The objective here is to ascertain how the same interaction within the same biomaterial deviates as energy levels increase or decrease. Additionally, in heavy ion therapy, the significance of how the energy of the selected heavy ion affects range, ionization, recoils, and other secondary interactions is underscored.

## 3. Result

### 3.1. Bragg Cure

**Figure 2:**
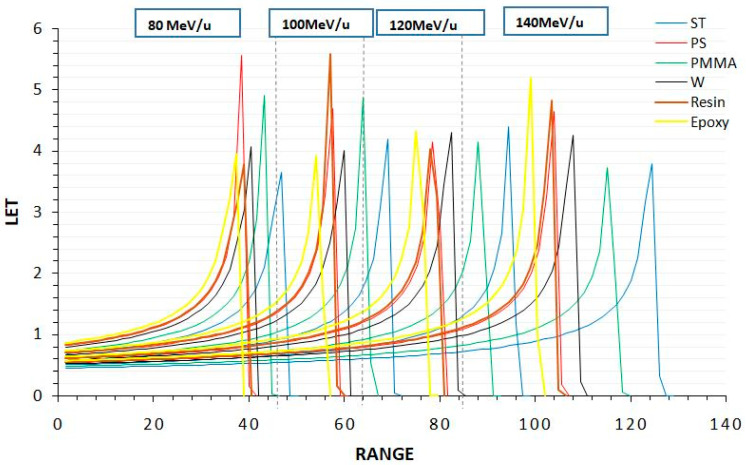
Profiles of LET (eV/A) and range (mm) for a ^7^Li ion beam within the energy range of 80–140 MeV/u in phantom materials.

In this study, the Bragg curve data obtained were evaluated. These data include Bragg curve profiles formed by ^7^Li ions with 80–140 MeV/u energy in different biomaterials (ST, water, PS, epoxy, resin and PMMA). The LET (eV/A) and range (mm) profiles of the Bragg curves are presented in Figure 2. Additionally, Bragg peak ranges, average ranges and deviations are given in Table 2. The data show that different biomaterials differ from each other in terms of Bragg peak ranges. Compared to ST, PMMA’s Bragg peak range is 7.3% lower, Resin 17.1% lower, epoxy 18.8% lower, water 13.1% and PS 16.8% lower. These data show that the Bragg peak locations increase by 22.5 mm, 24.0 mm, 21.9 mm, 25.9 mm, 21.5 mm, and 21.0 mm in water, PMMA, PS, ST, Resin and epoxy, respectively, for every 20 MeV/u increase in ^7^Li ion beam energy. In conclusion, these data show that the PMMA biomaterial is one of the closest biomaterials to ST.

**Figure 3:**
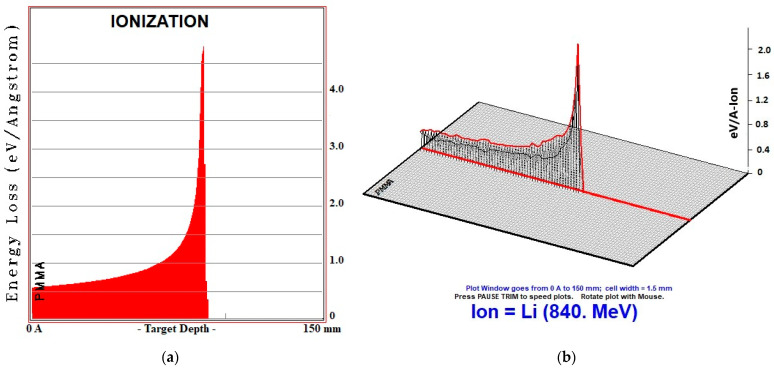
One-dimensional (**a**) and three-dimensional (**b**) ionization representations of PMMA biomaterial at 120 MeV/u energy.

One-dimensional (Figure 3a) and three-dimensional (Figure 3b) representations of PMMA biomaterial, which gives the closest ionization result to soft tissue because of MC TRIM calculations, are given in Figure 3.

### 3.2. Recoils

Table 3 presents data regarding the recoil peak generated by a ^7^Li ion beam within four distinct phantom materials, along with the corresponding percentages of constituent atoms contributing to this peak. The average recoil value in soft tissue (ST) was recorded as 2.537 eV/A across four different energy ranges. The contributing atoms to this recoil value include 2.35% hydrogen (H), 54.2% carbon (C), 20.1% oxygen (O), 0.2% chlorine (Cl), 20.5% magnesium (Mg), and 2.7% nitrogen (N). In the case of the water phantom, the average recoil across the four energy ranges was determined as 2.572 eV/A, with contributions from 35.8% H and 64.2% O. The polymethyl methacrylate (PMMA) phantom exhibited an average recoil value of 2.835 eV/A across the same energy ranges, with constituent atoms contributing as 30.4% H, 46.5% C, and 23.1% O.

In contrast, the resin phantom displayed an average recoil of 3.316 eV/A across the four energy ranges, with contributing atoms comprising 21.3% H, 55.9% C, 13.2% O, 3.9% Cl, 1.8% N, and 3.8% O. The ST phantom recorded an average recoil of 2.569 eV/A across the same energy ranges, with contributing atoms including 6.3% C, 7.1% O, 14.5% Cl, 11.2% Mg, 16.9% Ca, 9.9% F, 13.2% Si, 10.1% Na, and 10.8% O. Finally, the epoxy phantom exhibited an average recoil of 3.291 eV/A across the four energy ranges, with contributing atoms consisting of 19.3% H, 55.9% C, 13.4% O, and 6.6% Cl.

Notably, the soft tissue (ST) phantom displayed the closest total recoil value to that of the water phantom, with a difference of merely 1.4%, while the PMMA phantom exhibited a difference of 10.5% in comparison to ST tissue.

**Figure 4:**
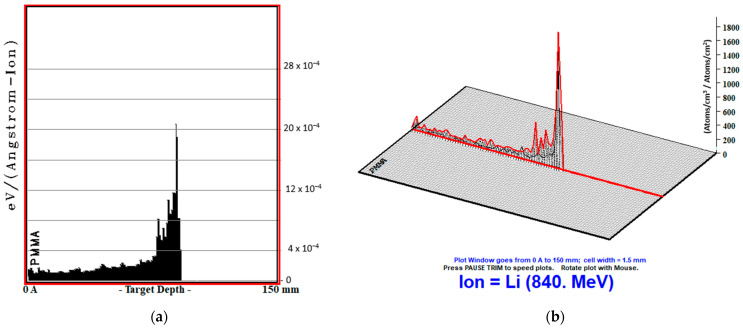
One-dimensional (**a**) and three-dimensional (**b**) recoil representations of PMMA biomaterial at 120 MeV/u energy.

**Figure 5:**
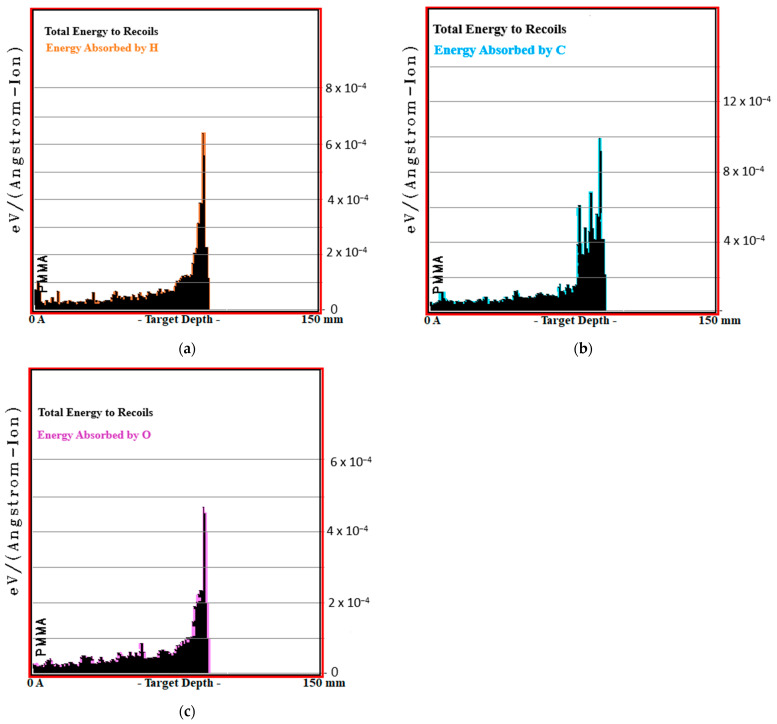
The percentages of constituent atoms forming the phantom and their corresponding standard deviations (S.D.) contributing to the Total Recoil (T.R.) value (eV/(Angstrom-Ion)) × 10^3^ of the ^7^Li ion beam within the energy range of 80–140 MeV/u in various phantoms are provided. (**a**) Energy Absorbed by H; (**b**) Energy Absorbed by C; (**c**) Energy Absorbed by O.

One- and three-dimensional MC TRIM simulation outputs of PMMA biomaterial, in which the ^7^Li ion beam interacts closest to the tissue, are given in Figure 4. The contribution energy graphs of the atoms (H, O and C) of the PMMA biomaterial contributing to this recoil value are given in Figure 5.

### 3.3. Collision Events

In this section, total CE created by ^7^Li ions and recoil interactions by colliding with atoms in ST, water, PMMA and PS phantoms are discussed. The gaps and displacements created by these CE in the target material are also examined. According to the data presented in Table 4, PS, as the closest biomaterial to ST, had the highest value in total target vacancies with a difference of 14.9%. Among the remaining biomaterials, PMMA came in second with a difference of 24.5%, while PE and water also differed from ST with differences of 54.0% and 54.0%, respectively. When the target material was examined in terms of total target displacements, the closest biomaterial to ST was again PS and had the highest total target displacement value with a difference of 15.6%. Among the remaining biomaterials, PMMA ranked second with a difference of 24.7%, while water differed from ST with a difference of 54.9%. When examined in terms of total target replacement collisions, the biomaterial closest to ST was PMMA, which had the highest total target replacement value, with a difference of 43.8%. Among the remaining biomaterials, PS took second place, with a difference of 85.4%, while water differed from ST with a difference of 141.6%. These results show that interactions of ^7^Li ions and recoils cause different vacancies, displacements and total target switching events in the target material. These findings can provide important information about the interaction of different materials with radiation in the biomedical field.

**Figure 6:**
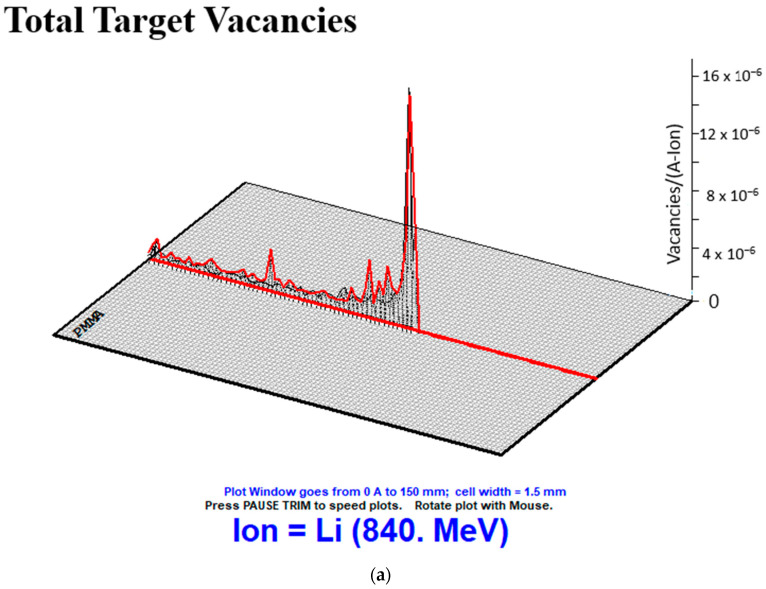

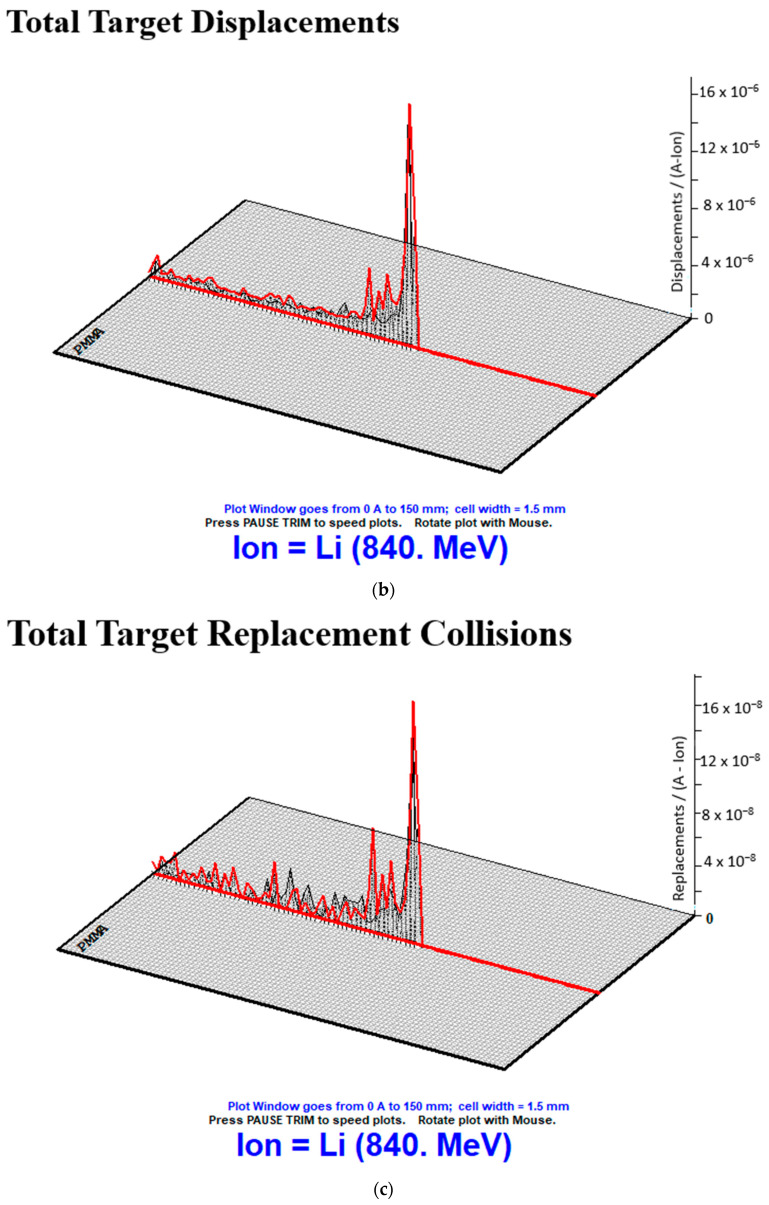
Images of total target vacancies, (**a**) total target displacements (**b**) and total target replacement collisions (**c**) created using 120 MeV/u energy Li bundle in PMMA biomaterial with the help of MC TRIM simulation.

With the help of MC TRIM simulation, the ^7^Li beam formed the PMMA biomaterial, which gives the closest ST values in CE interactions. The visuals of the total target vacancies, total target displacements and total target replacement collisions are given in Figure 6.

### 3.4. Lateral Straggle

**Figure 7:**
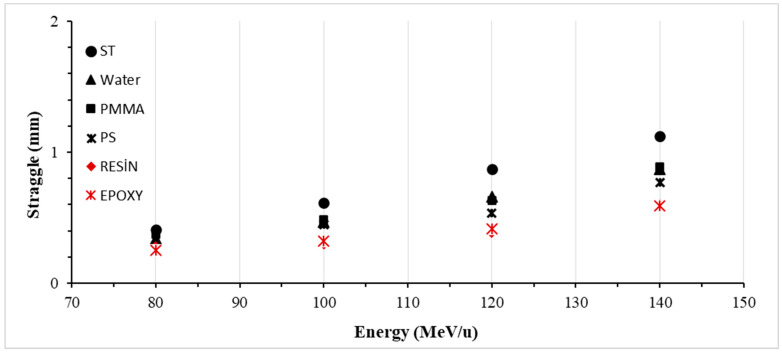
The LS arising from interactions between ^7^Li ion beams and various phantoms within the energy range of 80–140 MeV/u, occurring in the beam direction.

In this research study, we investigated the lateral straggle profiles of ^7^Li ions within the energy range of 80–140 MeV/u as they interacted with various phantom materials. The outcomes of experiments conducted on these phantoms are documented in Table 5 and illustrated in Figure 7. The mean lateral straggle value in the soft tissue (ST) phantom was determined to be 0.754 mm, showcasing a 36.6% increase from the smallest to the largest range. In the water phantom, the mean lateral straggle value measured 0.584 mm, with a 38.7% increase across the range. For the polymethyl methacrylate (PMMA) phantom, the mean lateral straggle value was calculated as 0.594 mm, demonstrating a 41.9% increase across the range. The mean lateral straggle value in the resin phantom was found to be 0.392 mm, indicating a 46.7% increase across the range. Similarly, in the epoxy phantom, the mean lateral straggle value was 0.396 mm, showing a 43.1% increase across the range.

Furthermore, we observed that among biomaterials, PMMA exhibited the lateral straggle values closest to those of soft tissue (ST), with a difference of 21.2%. Water showed a difference of 22.6%, polystyrene (PS) 32.2%, resin 48.1%, and epoxy 47.6%, respectively. These results provide valuable insights into the interactions of ^7^Li ions with different phantom materials, offering potential applications in the field of radiology. Particularly, we anticipate that further investigations using diverse phantoms will enhance our comprehension of the impact of radiation on biological tissues.

## 4. Discussion

In this study, Bragg peak parameters, recoils, phonon and lateral straggle values of the energetic ^7^Li ion used for therapeutic purposes were calculated using phantoms made of different tissue-equivalent biomaterials [8,35,36]. In previous similar studies, the radiological properties of heavy ions in different phantoms were calculated and presented. Such similar studies have been experimentally conducted at the Hyogo Ion Beam Medical Center (HIBMC) at the National Cancer Center [37], Kashiwa, Japan [38], and Massachusetts General Hospital, Boston, MA, USA [39]. Experimental research on heavy ion therapy is carried out in many different institutions around the world. However, all studies so far have focused on proton and carbon bundles [40,41]. In the future, ions other than carbon and protons are expected to be of therapeutic interest [42]. Therefore, it is very important to expand the application of heavy ion therapy so that other radiobiologically interesting heavy ions such as O, Li and N can be used for therapeutic purposes [1,11]. With imaginary experiments, it is possible to apply all therapeutically relevant ion species in heavy ion beams. However, they differ in properties, such as different heavy ion beams, biological efficacy, and lateral dose distribution [42].Therefore, dose distributions and LET (linear energy transfer) profiles may vary depending on the selected ion beam type [13]. In studies on various phantoms, the LET profiles of heavy ions such as H, He, Li, C and O in the phantom have been investigated [43]. Phantoms often contain tissue and organ simulations that differ depending on the location and firmness of the tumor [35,36]. These differences can be achieved through methods such as customizing the phantom material according to whether the tumor occurs in hard or soft tissue [8] or customizing the preferred type of heavy particle according to the location and depth of the tumor [1,11]. The efficacy of tumor therapy using heavy ion beams such as ^7^Li is currently of great interest [44]. As in this study, these heavy ions have been shown to be more effective in destroying target tissue in soft tissue models than particles with low LET due to their high LET properties and good calibration [38]. Therefore, heavy ions with high LET, such as ^7^Li ions, have been used successfully, especially in brain-based soft tissue tumors [45]. Therefore, the biological effects of radiology are still under investigation, especially in soft tissues such as the brain and vascular tissues [44]. As in this study, it is important to investigate the radiological properties of soft-tissue-equivalent materials [8].

In this study, it was stated that ions with high LET differ according to tumor location [1,11,36]. The phantom material may differ depending on whether the tumor is formed in hard or soft tissue [8], or the preferred type of heavy particle can be customized depending on the location and depth of the tumor [1,11]. Recently, heavy ion beams such as ^7^Li have attracted attention as they have high success rates in tumor treatment [44]. Heavy ions with high LET, such as ^7^Li ions suggested in this study, were particularly effective in brain-based soft tissue tumor treatments [43]. The hallmark of heavy ion therapy is the precise dose delivery to the tumor with a steep dose gradient that preserves the surrounding normal tissues [46]. Although the LET values of ^7^Li ions investigated in this study are lower than 12C [4,22], ^8^Be and ^10^B, they are higher than ^4^He and proton [1,8] ions. In addition, the deep dose and lateral dose distributions may differ between the ^7^Li ion and other ions [1,11,46]. Optimal ion therapy can be personalized depending on the characteristics of the target material [8,46]. Compared to proton beams, which strengthens its possible future clinical application, ions with higher LET, such as ^4^He, ^7^Li, ^8^Be, ^10^B and carbon ions, show a steeper lateral distribution [1,46]. ^7^Li beams require less LS compared to proton [46] and ^4^He beams and less energy compared to ^8^Be and ^10^B carbon ion beams [1,37]. The main goal of all radiation therapies is to protect the surrounding healthy tissue while delivering the maximum therapeutic dose of radiation to the target. Therefore, it is important to investigate LS [1,36]. In cases such as ^7^Li, which is a heavy ion beam, it has been observed that millimetric deviations in tissues are close to critical points in tumor treatment and may cause serious effects [8]. Therefore, range verification and calibration of the ion beam are of great importance and have been investigated using different biomaterials for different organs [1,8,37]. Ideally, it is preferable to use only a single phantom material for an experimental study. However, two polymeric materials were investigated in this study. The determination of the most suitable phantom material for tissues has also been studied in other studies [8]. With the development of heavy ion therapy systems, the search for tissue equivalence of phantom materials has become even more important. Interactions such as recoil, collision events, and LS also play an important role in biomaterial selection, but more research is needed to fully understand these interactions [1,11,36]. It is generally accepted that the correct result of the phantom is related to the tissue equivalence of the phantom building materials [8]. Investigation of this equivalence with different heavy ion beams is critical for the success of the treatment. Therefore, research using different phantom materials plays an important role in developing and optimizing heavy ion therapy.

## 5. Conclusions

The results obtained in this study provide a new understanding of the interactions of polymeric biomaterials with the ^7^Li ion beam. The TRIM MC method is a Monte Carlo method used in this study and models the interaction of many ions with matter. This method is a suitable choice for modeling the interactions of the ion beam with polymeric biomaterials. The two main interactions identified in the study are ionization and recoil interactions. Ionization interaction is the process by which the ion interacts with matter and electrons break off, forming an ionized gas. Recoil interaction, on the other hand, is the process in which the ion interacts with the matter, and some of its energy is transferred to the matter, but the direction of the ion does not change. The phonon production resulting from these interactions was also investigated. Phonons are vibrations in the crystalline structure and they are excited by ions. The results obtained were compatible with the literature. In addition, the crystal structure change properties of polymeric biomaterials when bombarded with ^7^Li ion beam were determined in this study through the use of CE parameters. These parameters are used to calculate the number of collisions and the effects of nuclear interactions. The obtained results were compared with similar studies in the literature and discussed. In conclusion, considering the results obtained in the study and the studies in the literature, the importance of the use of biomaterials close to tissues in radiotherapy has been revealed. Innovative aspects of this study include:

- Utilizing Li ions as intermediate ions in heavy ion therapy and examining them in terms of ionization, recoils, and lateral straggle.
- Conducting a radiological investigation of polymer biomaterials used in phantom production or as body soft tissue replacements with heavy ions.
- Analyzing the structure of crystalline injection zones in polymer materials and investigating changes in polymer bonds caused by heavy ions.

It is worth noting that, due to the absence of an experimental heavy ion facility in our country, this study has not been replicated experimentally. Therefore, it is recommended that similar studies be carried out that include different types of biomaterials. These calculations can also be performed for different heavy ions and provide a more comprehensive understanding of the effects of radiation.

## Figures and Tables

**Table 1:** Atomic and mass composition percentages/density, displacement, binding and surface of biomaterials.

Biomaterials	Atomic Percent	Mass Percent	Atomic Number Density	Mass Density	Displacement	Binding	Surface
ST	H 54.6; C 32.9; N 0.862; O 7.89; Mg 3.63; CI 1.72	H 8.12; C 58.3; N 1.78; O 18.6; Mg 13.0; CI 8.99	8.88	1.0	H 10; C 28; N 28; O 28; Mg 25; CI 25	H 3; C 3; N 3; O 3; Mg 3; CI 3	H 2; C 7.41; N 2; O 2; Mg 1.24; CI 2
W	O 33.3; H 66.6	O 88.8; H 11.1	10.02	1.0	O 28; H 10	O 3; H 3	O 2; H 2
PMMA	H 53.3; C 33.3; O 13.3	H 8.05; C 59.9; O 31.9	8.57	0.95	H 10; C 28; O 28	H 3; C 3; O 3	H 2; C 7.41; O 2
PS	H 50; C 50	H 7.74; C 92.2	9.81	1.06	H 10; C 28	H 3; C 3	H 2; C 7.41
Resin	C 42; N 1.3; O 7.9; P 1.3; CI 1.3; H 46	C 64; N 2.3; O 16.2; P 5.4; CI 5.9; H 6.2	8.52	1.11	C 28; N 28; O 28; P 25; CI 25; H 10	C 3; N 3; O 3; P 3; CI 3; H 3	C 7.41; N 2; O 2; P 3.27; CI 3; H 2
Epoxy	C 42.2; N 2.2; O 8.9; P 2.2; CI 2.3; H 42.2	C 58.1; N 3.6; O 16.3; P 7.9; CI 9.1; H 4.9	8.16	1.18	C 28; N 28; O 28; P 25; CI 25; H 10	C 3; N 3; O 3; P 3; CI 3; H 3	C 7.41; N 2; O 2; P 3.27; CI 2; H 2

**Table 2:** Determination of the Bragg peak in ST (soft tissue), W (water), PS (polystyrene), resin, epoxy, and PMMA (polymethyl methacrylate) phantoms for a ^7^Li ion beam within the energy range of 80–140 MeV/u, including measurements of ranges (in centimeters), averages, and standard deviations.

Energy	Bragg Peak Position (mm)					
	W	PMMA	PS	ST	Resin	Epoxy
80	40.5	43.2	38.4	46.8	39.1	36.1
100	60.2	64.1	57.6	69.1	57.2	54.2
120	82.5	88.2	78.4	94.5	78.1	75.2
140	108.1	115.2	104	124.5	103.5	99.2
Average	72.825	77.675	69.6	83.725	69.475	66.175
S.D.	25.21	26.89	24.38	28.97	24.01	23.56

**Table 3:** The percentages of constituent atoms forming the phantom and their corresponding standard deviations (S.D.) contributing to the Total Recoil (T.R.) value (eV/(Angstrom-Ion)) × 10^3^ of the ^7^Li ion beam within the energy range of 80–140 MeV/u in various phantoms are provided.

Materials	Energy	T.R.	Contributions to Recoils of Atoms (%)											
			H	C	O	Cl	Mg	N	P	Ca	F	Si	Na	Al
ST	80	3.04	2.4	57.9	20.5	0.2	18.3	1.5						
	100	3.31	2.3	51.8	19.5	0.2	20.2	7.1						
	120	1.31	2.5	53.3	19.3	0.1	24.3	1.5						
	140	2.49	2.3	54.6	21.5	0.2	20.5	1.9						
	S.D.	0.89	0.1	2.6	1.1	0.1	2.5	2.7						
Water	80	1.57	37.2		64.2									
	100	1.57	34.7		66.9									
	120	3.66	37.9		63.6									
	140	3.49	36.9		64.6									
	S.D.	1.16	1.4		1.4									
PMMA	80	3.77	25.3	53.6	22.4									
	100	2.86	30.7	46.1	24.7									
	120	2.13	39.6	40.8	20.7									
	140	2.59	27.7	48.5	25.3									
	S.D.	0.69	6.3	5.3	2.1									
PS	80	3.18	25.9	75.5										
	100	3.93	23.1	78.1										
	120	2.78	22.7	78.5										
	140	3.31	23.3	77.9										
	S.D.	0.48	1.5	1.4										
Resin	80	3.28	21.2	56.3	13.3	3.8		1.6	3.8					
	100	3.93	19.5	59.9	13.9	2.3		1.9	2.5					
	120	2.79	22.1	54.2	14.2	4.2		2.1	3.2					
	140	3.28	22.6	53.3	11.6	5.3		1.6	5.6					
	S.D.	0.47	1.4	2.9	1.2	1.3		0.2	1.3					
Epoxy	80	3.416	19.2	56.5	14.8	6.6								
	100	3.207	20.4	54.5	15.6	5.9								
	120	2.987	19.4	53.4	11.9	5.3								
	140	3.553	18.1	59.1	11.4	8.5								
	S.D.	0.247	0.9	2.5	2.1	1.4								

**Table 4:** The total CE count (Number/Angstrom) and its associated standard deviations (S.D.) within phantoms subjected to a ^7^Li ion beam within the energy range of 80–140 MeV/u.

CE	Biomaterials	Energy (MeV/u)				S.D.
		80	100	120	140	
Total Target Vacanies	Water	25,840,000	29,870,000	33,720,000	37,290,000	4,272,452
	PMMA	20,800,000	24,070,000	27,280,000	30,290,000	3,542,563
	PS	19,060,000	22,220,000	25,200,000	28,040,000	3,346,117
	ST	16,680,000	19,310,000	21,920,000	24,360,000	2,868,200
	Resin	19,780,000	22,970,000	26,010,000	28,960,000	3,419,481
	Epoxy	19,580,000	22,730,000	25,790,000	28,660,000	3,388,385
Total Target Displacements	Water	26,270,000	30,370,000	34,290,000	37,920,000	4,347,404
	PMMA	21,060,000	24,370,000	27,620,000	30,670,000	3,587,276
	PS	19,390,000	22,610,000	25,630,000	28,530,000	3,404,247
	ST	16,860,000	19,520,000	22,160,000	24,630,000	2,901,735
	Resin	19,880,000	23,090,000	26,140,000	29,110,000	3,437,372
	Epoxy	19,790,000	22,970,000	26,070,000	28,960,000	3,423,101
Total Target Replacement Collisions	Water	440,000	510,000	570,000	630,000	70,489
	PMMA	260,000	300,000	340,000	380,000	44,721
	PS	330,000	390,000	440,000	490,000	59,319
	ST	180,000	210,000	240,000	260,000	30,311
	Resin	100,000	120,000	130,000	150,000	18,028
	Epoxy	210,000	240,000	280,000	310,000	38,079

**Table 5:** The LS arises in the beam direction due to interactions between ^7^Li ions and various phantoms within the energy range of 80–140 MeV/u.

Energy	Lateral Straggle (mm)					
	Water	PMMA	PS	ST	Resin	Epoxy
80	0.337	0.373	0.287	0.412	0.279	0.254
100	0.467	0.483	0.450	0.615	0.302	0.322
120	0.661	0.631	0.536	0.872	0.389	0.416
140	0.870	0.890	0.772	1.120	0.597	0.591
Average	0.584	0.594	0.511	0.755	0.392	0.396
S.D.	0.233	0.224	0.202	0.308	0.145	0.146

## Data Availability

Data are contained within the article.
